# Supplementary figures and images for: Using canavanine resistance to measure mutation rates in Schizosaccharomyces pombe
Source: PLoS One. 2023 Jan 10;18(1):e0271016. doi: 10.1371/journal.pone.0271016 (PMC9831302; doi:10.1371/journal.pone.0271016)

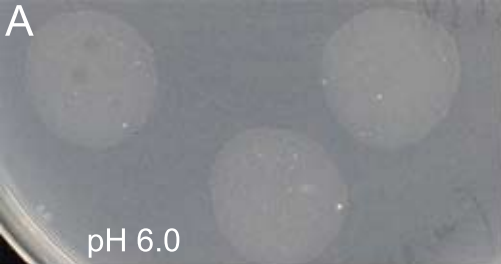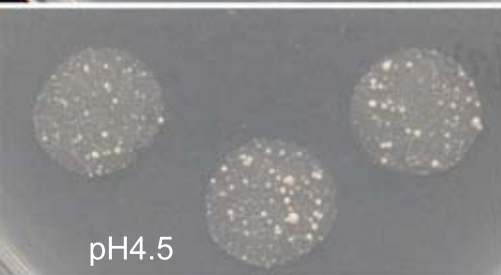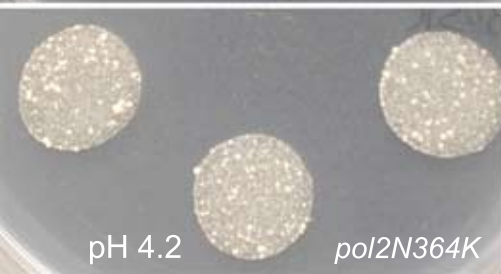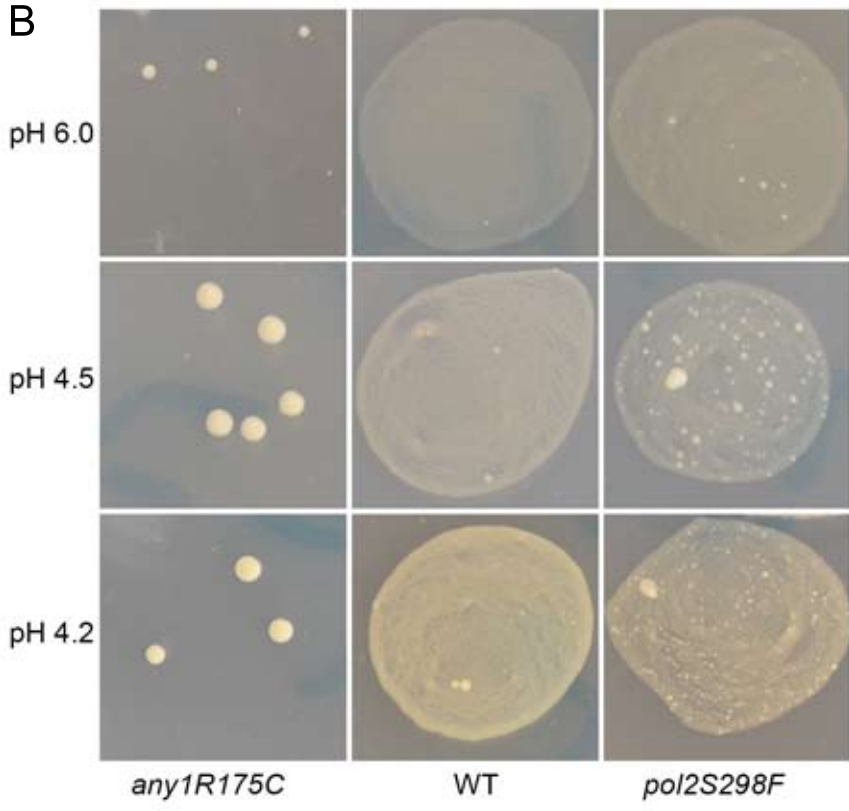

Supplement: S1 Fig — (A) 150 μl of a culture of strain 4355 (OD600 = 1) was spotted onto PMG + 80 μg/ml canavanine plates adjusted to the pHs shown. Plates were photographed after 5 days at 30°C. (B) As (A) except WT, any1R175C and pol2S298F strains were used. For the any1R175C strain approximately 5 cells were plated. (PDF) [file pone.0271016.s001.pdf]

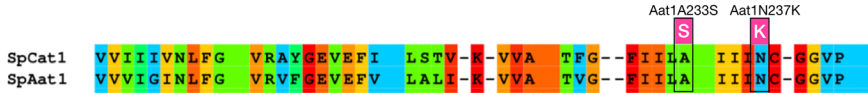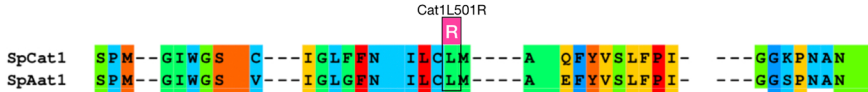

Unconserved 0 1 2 3 4 5 6 7 8 9 10 Conserved

Supplement: S2 Fig — Sequences aligned are S. pombe Cat1 and Aat1. The alignment shows that the missense mutations detected in some of the canavanine-resistant strains are conserved between Cat1 and Aat1. The alignment was generated by Praline [29]. (PDF) [file pone.0271016.s002.pdf]

766

1

2

3

4

5

6

7

500

300

150

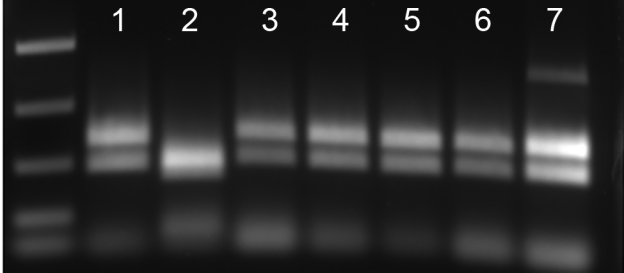

Supplement: S3 Fig — Strains were genotyped by amplifying an any1 gene fragment using oligos 1436 and 1437, followed by HpyCH4IV digestion. Lane 1, any1R175C control (strain 3647); lane 2, WT control (strain 2299); lanes 3–7, independent canavanine-resistant clones derived from hypermutating strain pol2S298F (3221). (PDF) [file pone.0271016.s003.pdf]
