# Supplementary material for: Using canavanine resistance to measure mutation rates in Schizosaccharomyces pombe
Source: PLoS One. 2023 Jan 10;18(1):e0271016. doi: 10.1371/journal.pone.0271016 (PMC9831302; doi:10.1371/journal.pone.0271016)
Supplement: S1 Table — (PDF) [file pone.0271016.s004.pdf]

Supplementary information

S1 Table. Strains used.

Strain number	Genotype	Reference/comments/source
2299	*h⁻*	972 (wt)
3188	*h⁻ pol2L425V::kanMX6 ade6-485*	This work
3199	*v pol2-D276A/E278A::kanMX6 ade6-485*	[1]
3202	*h⁻ pol2V412L::kanMX6 ade6-485*	This work
3221	*h⁻ pol2S298F::kanMX6 ade6-485*	This work
3647	*h- any1R175C pas1*	Originally "can1-1" (FY18665), YGRC, Japan
3786	*h⁻ cat1Δ::kanMX6 ade6-M210 ura4-D18 leu1-32*	Bioneer
3790	*h⁺ aat1Δ::ura4⁺*	Dr K. Takegawa
3791	*vhc1Δ::hphMX6 ura4*	This work
3796	*SPBPB2B2.01Δ::kanMX6 ade6-M210 ura4-D18 leu1-32*	Bioneer
3797	*aat1Δ::ura4⁺ cat1Δ::kanMX6*	This work
3938	*clr4Δ::kanMX6*	T. Humphrey
4059	*h- cat1⁺-GFP::kanMX6*	Dr A. Nakashima
4068	*h- any1R175C*	This work
4088	*any1R175C cat1⁺-GFP::kanMX6*	This work
4355	*h⁻ pol2N364K::kanMX6 ade6-485*	[2]

1. Aoude LG, Heitzer E, Johansson P, Gartside M, Wadt K, Pritchard AL, et al. POLE mutations in families predisposed to cutaneous melanoma. Fam Cancer. 2015;14(4):621-8. Epub 2015/08/08. doi: 10.1007/s10689-015-9826-8. PubMed PMID: 26251183.
2. Bertrand S. Defects in replicative DNA polymerases linked to cancer predisposition and tumour development: University of Oxford; 2020.
