# Supplementary material for: Using canavanine resistance to measure mutation rates in Schizosaccharomyces pombe
Source: PLoS One. 2023 Jan 10;18(1):e0271016. doi: 10.1371/journal.pone.0271016 (PMC9831302; doi:10.1371/journal.pone.0271016)
Supplement: S2 Table — (PDF) [file pone.0271016.s005.pdf]

S2 Table. Oligos used.

Oligo no.	5'>3'	Used for construction
1108	CTTTGACACCTTGACTTCCTTGAGGTAAATAAC	*pol2L425V*
1109	AAGGTGTCAAAGCTGTCACTGTCAGTAAATTAGG	*pol2L425V*
1137	CGGTGGCTTAAGAGAGATAGTTATTTACCTCAAG	*pol2V412L*
1138	TCTCTCTTAAGCCACCGGAAGGCATCCATAT	*pol2V412L*
1144	GATGATATTCTATATGATTGATGGCCAAGGA	*pol2S298F*
1145	TCAATCATATAGAATATCATCATTATTTTATCG	*pol2S298F*
1419	CGCCTGATACCATCCATAAAT	
1420	CGCCTGATACCATCCATAAAC	
1421	ATCCGACCCACATATTCTAGG	
1382	ATTTAATAACCTTGACAGTTCCTACCACAAAATTATTGCC TTCCTTATTTTATATTTAATGGGGATAATTTTTTGTGATT CGGATCCCCGGGTTAATTAA	
1383	CATGGACCAAATATTTAAATAGTTATAACAATGCCAAAAG AAAAGACTTTATAAACCAAAGACATAAATACCATAAATCG GAATTCGAGCTCGTTTAAAC	
1432	CTACACTCACTCTTCCTTACCATTTCC	
1433	GTTTTCGAGCTCCTATGAAATATATGCAAG	
1436	GGTGCACTTGCGTCTGGC	
1437	CCCGATTGTAAATCTTGTGTACTC	
