# Supplementary material for: Using canavanine resistance to measure mutation rates in Schizosaccharomyces pombe
Source: PLoS One. 2023 Jan 10;18(1):e0271016. doi: 10.1371/journal.pone.0271016 (PMC9831302; doi:10.1371/journal.pone.0271016)
Supplement: S3 Table — (PDF) [file pone.0271016.s006.pdf]

S3 Table. Absolute mutation rates of Pol2 strains

Absolute mutation rates									
Strain	ADE			CAN			FOA		
	Mutation Rate	Upper Bound	Lower Bound	Mutation Rate	Upper Bound	Lower Bound	Mutation Rate	Upper Bound	Lower Bound
POLE WT	0.9×10^{-8}	1.4	0.5	1.8×10^{-8}	2.6	1.1	1.5×10^{-8}	2.0	1.1
POLE D276E/A278E	2.3×10^{-8}	3.4	1.3	8.2×10^{-8}	11.9	5.0	21.5×10^{-8}	27.1	16.4
POLE S298F	12.1×10^{-8}	15.9	8.8	14.2×10^{-8}	19.2	9.9	72.4×10^{-8}	84.8	60.6
POLE V412L	1.8×10^{-8}	2.7	1.1	4.0×10^{-8}	5.7	2.5	7.0×10^{-8}	9.2	5.0
POLE L425V	1.7×10^{-8}	2.6	0.9	4.4×10^{-8}	7.0	2.2	10.8×10^{-8}	13.2	8.5
POLE S460F	7.2×10^{-8}	10.3	4.4	23.9×10^{-8}	31.1	17.5	62.3×10^{-8}	76.0	49.7
