# Supplementary material for: Using canavanine resistance to measure mutation rates in Schizosaccharomyces pombe
Source: PLoS One. 2023 Jan 10;18(1):e0271016. doi: 10.1371/journal.pone.0271016 (PMC9831302; doi:10.1371/journal.pone.0271016)
Supplement: S1 Raw data — (PDF) [file pone.0271016.s007.pdf]

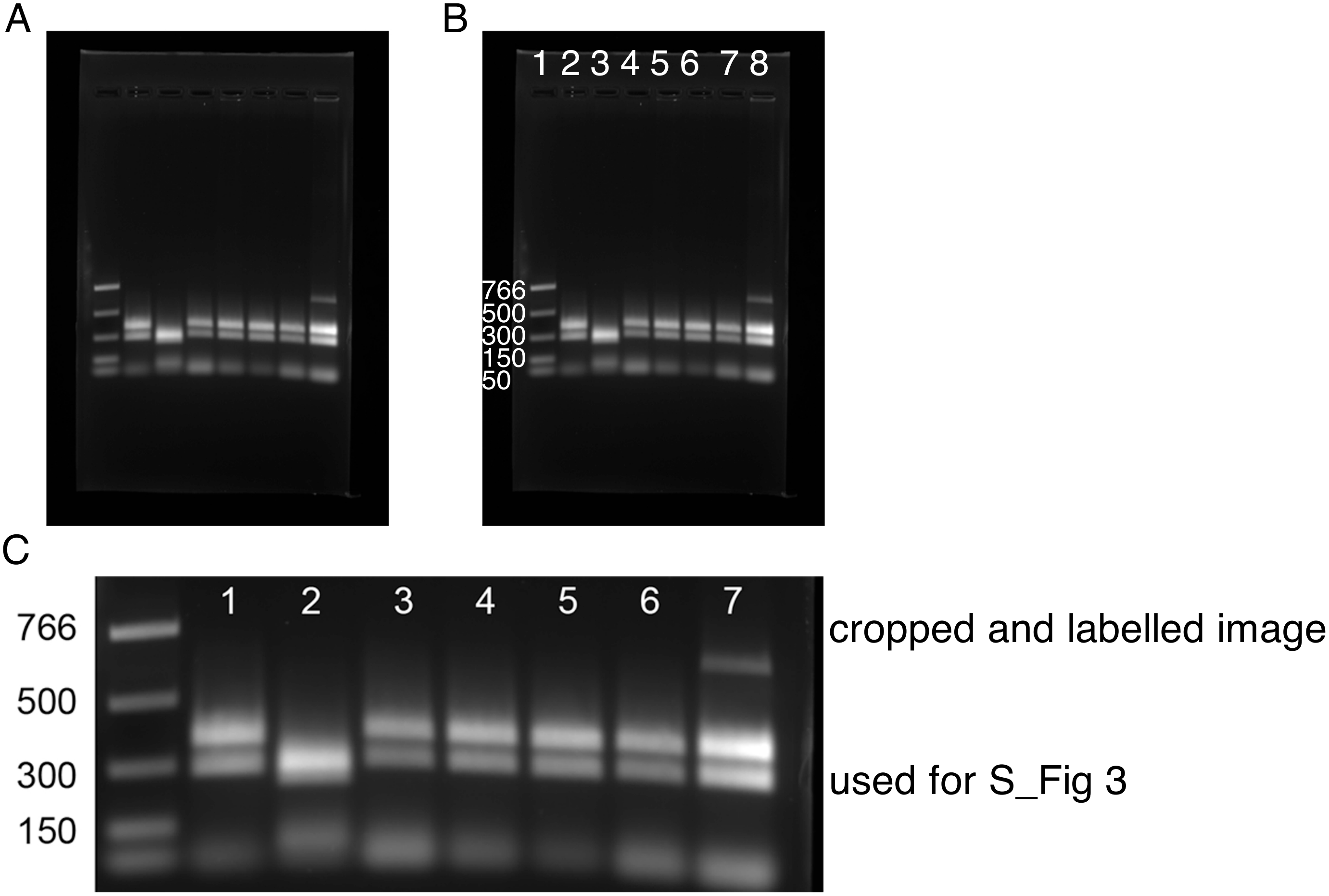

Fig S1_raw_datav2.pdf

A) Original gel image. 1.5% agarose Tris-acetate EDTA gel. DNA was visualised by Midori Green staining and image was taken on an iBright imaging system.

B) As (A) except lanes are labelled: 1 MW marker (bp sizes indicated); 2-8 Strains were genotyped by PCR amplifying an any1 gene fragment using oligos 1436 and 1437, followed by HpyCH4IV digestion, lane 2, any1R175C control (strain 3647); lane 3, WT control (strain 2299); lanes 4-8, independent canavanine-resistant clones derived from hypermutating strain pol2S298F (3221).

C) Cropped and labelled image used for Fig S3.
